# Contrasting effects of ascorbate and iron on the pulmonary vascular response to hypoxia in humans

**DOI:** 10.14814/phy2.12220

**Published:** 2014-12-11

**Authors:** Nick P. Talbot, Quentin P. Croft, M. Kate Curtis, Brandon E. Turner, Keith L. Dorrington, Peter A. Robbins, Thomas G. Smith

**Affiliations:** 1 Department of Physiology, Anatomy and Genetics, University of Oxford, Oxford, U.K; 2 Nuffield Division of Anaesthetics, John Radcliffe Hospital, University of Oxford, Oxford, U.K

**Keywords:** Ascorbate, hypoxia‐inducible factor, hypoxic pulmonary vasoconstriction, iron, pulmonary circulation, vitamin C

## Abstract

Hypoxia causes an increase in pulmonary artery pressure. Gene expression controlled by the hypoxia‐inducible factor (HIF) family of transcription factors plays an important role in the underlying pulmonary vascular responses. The hydroxylase enzymes that regulate HIF are highly sensitive to varying iron availability, and iron status modifies the pulmonary vascular response to hypoxia, possibly through its effects on HIF. Ascorbate (vitamin C) affects HIF hydroxylation in a similar manner to iron and may therefore have similar pulmonary effects. This study investigated the possible contribution of ascorbate availability to hypoxic pulmonary vasoconstriction in humans. Seven healthy volunteers undertook a randomized, controlled, double‐blind, crossover protocol which studied the effects of high‐dose intravenous ascorbic acid (total 6 g) on the pulmonary vascular response to 5 h of sustained hypoxia. Systolic pulmonary artery pressure (SPAP) was assessed during hypoxia by Doppler echocardiography. Results were compared with corresponding data from a similar study investigating the effect of intravenous iron, in which SPAP was measured in seven healthy volunteers during 8 h of sustained hypoxia. Consistent with other studies, iron supplementation profoundly inhibited hypoxic pulmonary vasoconstriction (*P* < 0.001). In contrast, supraphysiological supplementation of ascorbate did not affect the increase in pulmonary artery pressure induced by several hours of hypoxia (*P* = 0.61). We conclude that ascorbate does not interact with hypoxia and the pulmonary circulation in the same manner as iron. Whether the effects of iron are HIF‐mediated remains unknown, and the extent to which ascorbate contributes to HIF hydroxylation in vivo is also unclear.

## Abstract (precis)

We report here that intravenous ascorbate (vitamin C) supplementation had no effect on the increase in pulmonary artery pressure associated with several hours in a hypoxia chamber, whereas intravenous iron supplementation profoundly inhibited this response. It is not known whether the pulmonary effects of iron are mediated by hypoxia‐inducible factor (HIF), which plays an important role in pulmonary vascular responses to hypoxia and is sensitive to varying iron and ascorbate availability.

## Introduction

In previous studies we have established that pulmonary vascular responses to hypoxia are attenuated by iron supplementation and enhanced by iron depletion. In a laboratory study, the elevation in pulmonary artery pressure observed following sustained (8 h) hypoxia, and the accompanying increase in pulmonary vascular sensitivity to acute (20 min) hypoxia, were both blunted by prior administration of intravenous iron (Smith et al. 2008a). In contrast, reducing iron availability with intravenous desferrioxamine had opposite pulmonary effects (Smith et al. 2008a). In a subsequent field study at high altitude, loading with intravenous iron rapidly reversed the pulmonary hypertensive response resulting from 3 days of hypoxia, whereas reducing iron availability in chronically hypoxic residents of high altitude, using progressive venesection over 4 days, caused an increase in pulmonary artery pressure (Smith et al. 2009).

While the mechanism underlying this interaction between iron, hypoxia, and the pulmonary circulation is unknown, it is likely that the hypoxia‐inducible factor (HIF) family of transcription factors is involved. HIF regulates the expression of more than 2% of the human genome and plays a central role in maintaining oxygen homeostasis both intracellularly and at the systemic level (Manalo et al. 2005; Smith et al. 2008b; Semenza 2009). Hypoxia increases pulmonary artery pressure through hypoxic pulmonary vasoconstriction and subsequent vascular remodeling, and HIF is known to be important in these processes (Barbera et al. 2003; Shimoda and Laurie 2014). In humans, rare HIF‐activating mutations are associated with pulmonary hypertension (Bushuev et al. 2006; Smith et al. 2006; Gale et al. 2008; Bond et al. 2011) and with extremely high hypoxic pulmonary vasoreactivity (Smith et al. 2006, 2008c; Formenti et al. 2011), such that even the mild cabin hypoxia experienced during an airline flight can lead to transient pulmonary hypertension (Smith et al. 2012, 2013). In genetically engineered mice, HIF‐activating mutations lead to comparable effects (Hickey et al. 2010; Tan et al. 2013), while, conversely, partial inactivation of HIF inhibits pulmonary vascular responses to hypoxia (Yu et al. 1999; Shimoda et al. 2001, 2006; Brusselmans et al. 2003; Wang et al. 2006; Ball et al. 2014).

Hypoxia‐inducible factor is primarily regulated through its oxygen‐dependent proteosomal degradation (Epstein et al. 2001). In addition to conferring the ability to sense and respond to hypoxia, this proteosomal degradation is also dependent on iron and ascorbate (vitamin C) availability, as these are obligate cofactors in the key oxygen‐sensitive hydroxylation step that is catalyzed by HIF hydroxylase enzymes (Ivan et al. 2001; Jaakkola et al. 2001; Yu et al. 2001). In cultured cells, HIF degradation is respectively potentiated and inhibited by increasing and decreasing iron availability (Wang and Semenza 1993; Knowles et al. 2003), and this could account for the effects of iron on pulmonary vascular responses to hypoxia. Importantly, varying ascorbate availability has similar effects on HIF in cultured cells (Knowles et al. 2003), and ascorbate may therefore affect the lungs in a similar manner. Such an effect could have implications for patients with pulmonary arterial hypertension, in whom promising clinical trials are now underway investigating the therapeutic use of intravenous iron (Rhodes et al. 2011; Howard et al. 2013). Were ascorbate to have similar pulmonary effects, it could potentially present a safe and convenient treatment option in this disease.

This study aimed to determine whether intravenous loading with ascorbate inhibits the increase in pulmonary artery pressure caused by hypoxia. Sequential measurements of systolic pulmonary artery pressure (SPAP) were made by Doppler echocardiography during 5 h of sustained hypoxia with and without ascorbate loading. Sequential measurements of SPAP during sustained hypoxia are also presented from an earlier laboratory study with and without intravenous iron. That study focussed on extensive measurements before and after 8 h inside a hypoxia chamber (Smith et al. 2008a). Data collected during that iron study within the chamber itself have not previously been published, and are included here to allow comparison with the corresponding data from the present ascorbate study.

## Materials and Methods

### Ascorbate study

This study tested the hypothesis that increasing ascorbate availability would attenuate the elevation in SPAP that is normally caused by sustained exposure to hypoxia. The protocol used a randomized, controlled, double‐blind, crossover design. Each participant underwent an ascorbate day (when they received ascorbate) and a control day (when they received saline as a placebo) in random order. The two study days were identical except for the infusions and were separated by 2 weeks. Participants were taking no medications or supplements and had no recent history of travel to high altitude. The South Central – Oxford B Research Ethics Committee approved the study and all participants provided written informed consent.

Seven healthy volunteers participated in the study (three men and four women; Table 1). Experiments were conducted at the Altitude Centre, London, where a large room functions as a normobaric altitude chamber. Participants underwent a 5‐h period of sustained hypoxia in this study, during which the concentration of oxygen in the chamber was maintained at 13–14%.

**Table 1.** Participant characteristics.

Characteristics (normal range)	Ascorbate study	Iron study
*n*	7	7
Age (years)	25 ± 3	24 ± 3
Weight (kg)	64 ± 9	69 ± 12
Height (m)	1.75 ± 0.10	1.74 ± 0.05
Hemoglobin (12–17 g/dL)	14.8 ± 1.5	14.0 ± 1.6
Hematocrit (0.36–0.50 L/L)	0.43 ± 0.04	0.42 ± 0.04
Serum iron (11–31 *μ*mol/L)	19 ± 9	16 ± 6
Transferrin (1.8–3.6 g/L)	2.8 ± 0.3	2.6 ± 0.3
Transferrin saturation (16–50%)	30 ± 15	30 ± 12
Serum ferritin (10–300 *μ*g/L)	74 ± 67	82 ± 83

Mean ± SD values are shown. Where normal ranges vary with sex, the widest range is given.

The primary outcome measure was the change in SPAP measured by Doppler echocardiography during exposure to 5 h of poikilocapnic hypoxia. SPAP was determined using the standard Doppler technique used in our previous studies. Vivid‐*i* and Vivid‐*q* echocardiography machines (GE Medical Systems, Chalfont St Giles, Buckinghamshire, U.K.) were used to determine the maximum systolic pressure gradient across the tricuspid valve and SPAP was calculated using the modified Bernoulli equation and an estimated right atrial pressure of 5 mmHg (Yock and Popp 1984; Allemann et al. 2000; Balanos et al. 2005; Smith et al. 2008a, 2009). Cardiac output was also determined echocardiographically.

Two intravenous doses of ascorbic acid (Pascoe GmbH, Giessen, Germany) were administered: 3 g prior to entering the hypoxia chamber and 3 g at the half‐way point of hypoxia (2.5 h). This dosing schedule was based on the pharmacokinetics of intravenous ascorbate, which is quite rapidly eliminated (Muhlhofer et al. 2004; Padayatty et al. 2004). The ascorbate was constituted in 30 mL of normal saline and infused over 10 min. Control infusions consisted of 30 mL of normal saline infused over 10 min.

Heart rate, arterial oxygen saturation (SpO_2_), SPAP and cardiac output were measured immediately before and after the initial infusion (before entering the chamber), then hourly during the period of sustained hypoxia, and again after exiting the chamber. Blood pressure and ventilation (measured by Wright's respirometer [nSpire Health Ltd, Hertford, U.K.]) were measured immediately before and after the initial infusion (before entering the chamber), at the end of the period of sustained hypoxia, and again after exiting the chamber. Hemoglobin, hematocrit, and iron status were determined at baseline. Plasma concentrations of ascorbate, erythropoietin, and hepcidin were measured at the beginning and the end of the protocol on each day by ELISA, according to the respective manufacturer's instructions (ascorbate: Cusabio Biotech Ltd, Wuhan, China; erythropoietin: R&D systems, Minneapolis, MN; and hepcidin: Bachem, Bubendorf, Switzerland).

### Iron study

The methods for this study have been described previously (Smith et al. 2008a). Full echocardiographic measurements were possible in seven participants in the chamber. Briefly, healthy iron‐replete volunteers (four men and three women; Table 1) underwent an 8‐h period of isocapnic hypoxia with and without prior loading with intravenous iron (iron sucrose 200 mg; Syner‐Med Pharmaceutical Products Ltd, Purley, U.K.). The study was conducted in a purpose‐built hypoxia chamber in Oxford (Howard et al. 1995). End‐tidal partial pressure of oxygen was maintained at 55 mmHg, and end‐tidal partial pressure of carbon dioxide was maintained close to each participant's baseline value. The same echocardiographic technique used in the ascorbate study was used to measure SPAP and cardiac output before, during (hourly) and after the period of hypoxia. The Oxfordshire Clinical Research Ethics Committee approved the study and all participants provided written informed consent. While the ascorbate and iron studies differed in some respects (the ascorbate study used 5 h of poikilocapnic hypoxia, while the iron study used 8 h of isocapnic hypoxia), the design of each respective protocol was intended to generate a similar hypoxia‐induced increase in SPAP, allowing comparisons between the results.

### Statistical analyses

Student's paired *t* tests were used to compare differences in means. The effect of the infusions was assessed using a two‐factor repeated measures analysis of variance (ANOVA), where the within‐subject factors were time and the infusion (SPSS, Chicago, IL). Differences were considered significant at the *P* < 0.05 level. All values are expressed as means ± SD.

## Results

### Ascorbate study

Baseline characteristics of participants are shown in Table 1. All infusions were well tolerated. No statistically significant effects of ascorbate infusion were observed for any of the variables measured in this study.

**Figure 1.**
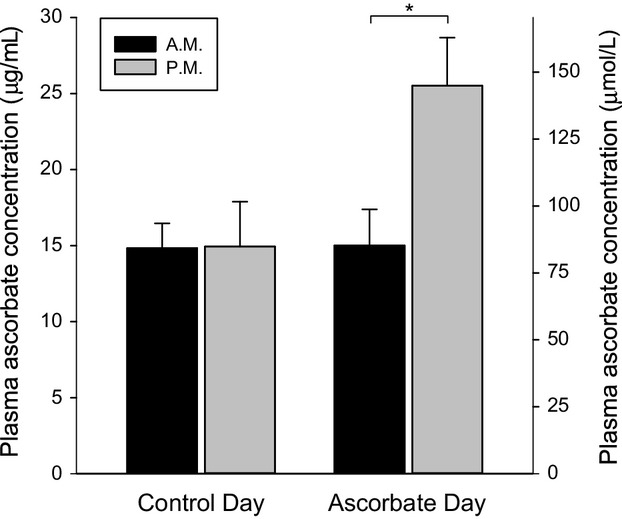
Plasma ascorbate concentration before and after infusion of saline control or ascorbate. Venous blood samples were taken at the beginning (denoted A.M.) and end (denoted P.M.) of the protocol on each day. *indicates a statistically significant difference (*P* < 0.001). Data are mean ± SD.

**Figure 2.**
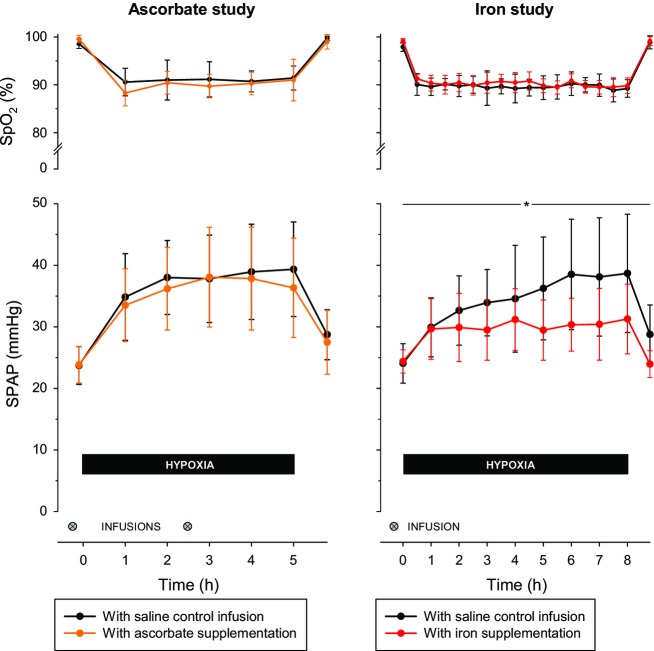
Arterial oxygen saturation and systolic pulmonary artery pressure during sustained hypoxia. Upper panels show arterial oxygen saturation (SpO_2_) and lower panels show systolic pulmonary artery pressure (SPAP) in the Ascorbate study and in the Iron study. The pulmonary vascular response to sustained hypoxia was not affected by increased ascorbate availability but was profoundly blunted by increased iron availability. This effect of iron became evident at the 2‐h time point (*P* < 0.05). *indicates a statistically significant overall effect of iron supplementation (*P* < 0.001). Data are mean ± SD.

**Figure 3.**
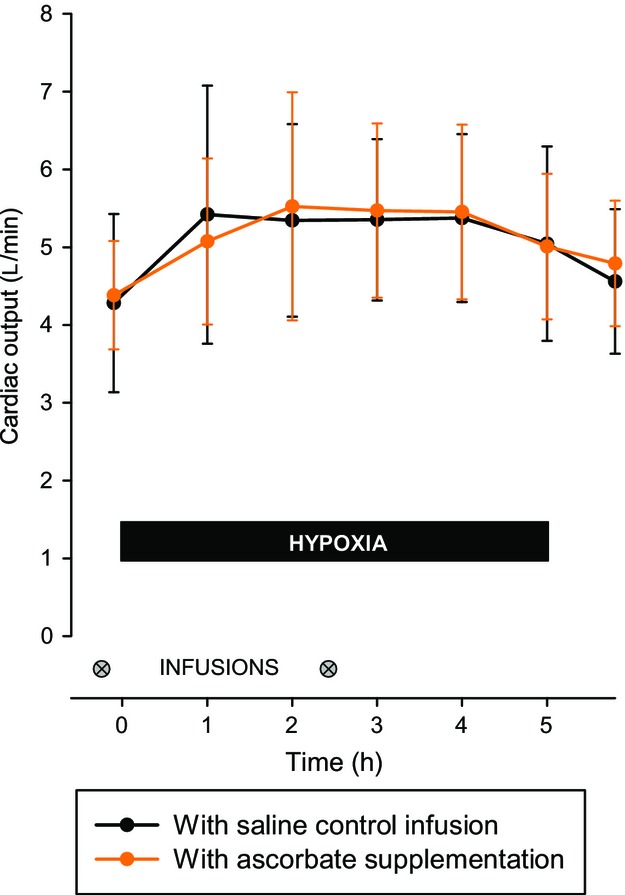
Cardiac output during sustained hypoxia with and without ascorbate loading. The cardiac output response to hypoxia was not affected by increased ascorbate availability. Data are mean ± SD.

Following the ascorbate infusions, plasma ascorbate concentration was substantially higher and exceeded the normal range of 2–20 *μ*g/dL (11–114 *μ*mol/L) (*P* < 0.001; Fig. 1). When measured immediately before and after the initial infusion, SPAP did not change significantly and was not affected by ascorbate (change in SPAP of −4 ± 5% with saline vs. −1 ± 4% with ascorbate, *P* = 0.29). Figure 2 shows that the reduction in SpO_2_ during sustained hypoxia was very similar on the two study days. Hypoxia induced a large increase in SPAP that was not affected by loading with ascorbate (compared with baseline, mean SPAP during hypoxia was increased by 55 ± 20% with saline control vs. 51 ± 20% with ascorbate, *P* = 0.61; Fig. 2). The increase in cardiac output induced by hypoxia was likewise unaffected by ascorbate (mean increase in cardiac output of 27 ± 22% with saline control vs. 22 ± 22% with ascorbate, *P* = 0.71; Fig. 3). Baseline ventilation prior to hypoxia was similar on the 2 days (13 ± 7 L/min on the control day vs. 11 ± 5 L/min on the ascorbate day, *P* = 0.33) and the increase in ventilation caused by hypoxia was not significantly affected by ascorbate (increase of 15 ± 23% with saline control vs. 38 ± 49% with ascorbate, *P* = 0.21).

The initial plasma erythropoietin concentration measured prior to the infusions was not significantly different on the two experimental days (6.5 ± 2.1 mIU/mL prior to control infusion and 5.7 ± 2.0 mIU/mL prior to ascorbate infusion, *P* = 0.28). The elevation in plasma erythropoietin concentration generated by hypoxia was not significantly affected by ascorbate (increase of 49 ± 25% with placebo vs. increase of 60 ± 43% with ascorbate, *P* = 0.91). Plasma hepcidin concentration was measured at the same time points. Baseline hepcidin levels were not significantly different on the two experimental days (23 ± 12 ng/mL prior to control infusion and 20 ± 19 ng/mL prior to ascorbate infusion; *P* = 0.63). Hypoxia did not cause a statistically significant change in hepcidin on either day (change of +36 ± 57% on the control day, *P* = 0.20; change of +53 ± 102% on the ascorbate day, *P* = 0.12).

### Iron study

Baseline characteristics of participants are shown in Table 1. When measured at the end of the day following iron infusions, indices of iron status were substantially higher than at baseline, with serum iron 63 ± 9 *μ*mol/L, transferrin saturation 108 ± 2% and serum ferritin 130 ± 82 *μ*g/L (all *P* < 0.001), while serum transferrin was unchanged (2.7 ± 0.4 g/L; *P* = 0.23). The fall in SpO_2_ during sustained hypoxia was very similar to that of the ascorbate study (Fig. 2). The rise in SPAP caused by hypoxia was inhibited by pretreatment with iron (compared with baseline, mean SPAP during hypoxia was increased by 30 ± 13% with saline control vs. 18 ± 10% with iron, *P* < 0.001; Fig. 2). This effect was evident after 2 h in the chamber (*P* < 0.05), and by the 5‐h time point the hypoxia‐induced increase in SPAP following iron infusion (increase in SPAP of 20 ± 14%) was less than half that observed following saline infusion (increase in SPAP of 50 ± 24%, *P* < 0.001). There was no difference between baseline cardiac output on the saline day (4.4 ± 1.1 L/min) and the iron day (4.4 ± 0.6 L/min; *P* = 0.85), and iron had no effect on the increase in cardiac output caused by hypoxia (mean increase in cardiac output of 17 ± 19% with saline control vs. 22 ± 25% with ascorbate, *P* = 0.23).

## Discussion

The effect of iron status on hypoxic pulmonary vasoconstriction, together with the biochemistry of HIF regulation and its involvement in pulmonary physiology, suggest that ascorbate availability may modify the effects of hypoxia on the pulmonary circulation. This study has not explored the effect of reduced ascorbate availability, but has established that, unlike iron supplementation, supraphysiological supplementation of ascorbate does not affect the increase in pulmonary artery pressure caused by several hours of hypoxia.

The HIF hydroxylases are members of a large class of enzymes known as 2‐oxoglutarate‐dependent dioxygenases, which catalyze the incorporation of oxygen into organic substrates through a mechanism that requires 2‐oxoglutarate, iron (Fe^2+^) and, in many cases, ascorbate. In humans this is exemplified by scurvy, in which ascorbate deficiency causes defective collagen formation by inhibiting one such enzyme, the collagen prolyl hydroxylase, leading to the characteristic signs of poor wound healing, bleeding gums, and widespread hemorrhages (Smith and Talbot 2010). According to multiple case reports, some patients with scurvy also develop severe pulmonary hypertension that rapidly resolves upon treatment with ascorbate, and that could in theory derive from inhibition of HIF hydroxylase enzymes (Mehta et al. 1996; Nagamatsu et al. 2009; Mertens and Gertner 2011; Kupari and Rapola 2012; Duvall et al. 2013).

However, the current study found no evidence for an interaction between ascorbate and hypoxic pulmonary vasoconstriction. One challenge in interpreting these findings, and in this field of research generally, is that the physiological contribution of ascorbate to HIF hydroxylation is not entirely clear. HIF hydroxylation is submaximal without ascorbate supplementation in isolated enzymes (Jaakkola et al. 2001; Hirsila et al. 2003; Flashman et al. 2010) and across many types of cultured cells, in which increasing ascorbate concentrations promote HIF hydroxylation and thereby inhibit HIF activation in a dose‐dependent manner (Knowles et al. 2003, 2006; Vissers and Wilkie 2007; Vissers et al. 2007; Kuiper et al. 2014). However, it has been suggested that these in vitro findings may reflect ascorbate deficiency in the culture medium to some extent, and they are generally not consistent with the few in vivo studies conducted to date. Parenteral high‐dose ascorbate has been shown to suppress testosterone‐induced HIF induction in rats (Li et al. 2010), yet hypoxia‐induced expression of erythropoietin was not affected by severe ascorbate deficiency in genetically engineered mice (Nytko et al. 2011), or by modest oral ascorbate supplementation in rats (Martinez‐Bello et al. 2012). The current study also found no effect of ascorbate on the hypoxia‐induced rise in plasma erythropoietin, which is well known to be mediated by HIF. Together these findings suggest that in vivo HIF hydroxylation may not necessarily be limited by physiological levels of ascorbate in humans, and thus may not be potentiated by supraphysiological supplementation. It should be noted though that supplemental iron likewise had no effect on the erythropoietin response to hypoxia (Smith et al. 2008a), yet profoundly inhibited the pulmonary vascular response.

It is not known how iron modifies hypoxic pulmonary vasoreactivity. Our primary hypothesis – that iron interferes with gene expression in the pulmonary vasculature via HIF – is supported by the time‐course of the response reported here. Hypoxic pulmonary vasoconstriction consists of two temporal components – an initial phase, in which pulmonary artery pressure plateaus after several minutes, and a subsequent intensification which begins after approximately 45 min and causes a progressive increase in pulmonary artery pressure (Talbot et al. 2005). The latency of this second phase has been attributed to new gene expression triggered by hypoxia, and it is this second phase that was blunted by iron loading; the initial reflex response was preserved.

It is of course possible that the striking pulmonary effects of iron are not mediated by HIF, and this could explain the lack of effect of ascorbate. Alternative theoretical explanations include a possible effect of iron on the formation of oxygen‐free radicals, or possible involvement of some unknown iron‐dependent oxygen sensor such as another member of the dioxygenase enzyme family to which the HIF hydroxylases belong (Smith et al. 2008a, 2009). A further possibility is that the pulmonary effects of iron are indeed mediated by HIF but through a mechanism that is independent of HIF hydroxylation (Ghosh et al. 2013). Irrespective of the precise underlying mechanism, clinical trials are currently seeking to take advantage of these effects using intravenous iron in patients with pulmonary arterial hypertension, a devastating disease in which novel therapies are urgently required (Rhodes et al. 2011; Howard et al. 2013). Unfortunately, the current data do not support similar clinical trials of intravenous ascorbate.

The lack of effect of ascorbate is unlikely to have resulted from technical limitations of the study. A typical daily dose of ascorbic acid for the treatment of scurvy is 1 g. The dose of ascorbate administered in this study, 3 g, was very high, and was repeated half‐way through the hypoxic period to ensure that plasma availability remained high, which was confirmed on repeat sampling. The protocols in the ascorbate study and the iron study were similar, with an equivalent severity of hypoxia that resulted in a substantial rise in SPAP in both studies. Each study used a robust controlled design, and while the two protocols were not identical, this is very unlikely to account for the contrasting results. The ascorbate study used a poikilocapnic protocol whereas the iron study used an isocapnic protocol, but the increase in SPAP was nevertheless comparable in the two studies, and in any case the effect of intravenous iron has also been observed with poikilocapnic hypoxia (Smith et al. 2009). Also, although the period of hypoxia was shorter in the ascorbate study (5 h) than in the iron study (8 h), the effect of iron was already evident after just 2 h, and after 5 h iron had more than halved the normal SPAP response to hypoxia.

We conclude that the pulmonary vascular response to several hours of hypoxia does not depend on ascorbate status in the same way it depends on iron status. Further research is required to determine whether altered HIF hydroxylation is responsible for the effect of iron, and whether the contrasting pulmonary effects we have observed arise from contrasting effects of ascorbate and iron availability on HIF hydroxylation in vivo.

## Acknowledgments

The authors thank the director of the Altitude Centre, R. Pullan, and staff for their cooperation, and the volunteers who took part in this study.

## Conflict of Interest

The authors declare that they have no conflicts of interest.
